# Multi-omics analysis identifies a hepatocyte-associated signature in alcohol-related liver injury

**DOI:** 10.3389/fimmu.2026.1844110

**Published:** 2026-07-22

**Authors:** Ran Ding, Fan Qi, Qianqian Dai, Kangfan Li, Yuan Zhang

**Affiliations:** 1 School of Biomedical Sciences and Engineering, South China University of Technology, Guangzhou International Campus, Guangzhou, China; 2 National Engineering Research Center for Tissue Restoration and Reconstruction, South China University of Technology, Guangzhou, China; 3 Guangdong Province Key Laboratory of Biomedical Engineering, South China University of Technology, Guangzhou, China; 4 Key Laboratory of Biomedical Materials of the Ministry of Education, South China University of Technology, Guangzhou, China

**Keywords:** alcohol-related liver disease (ALD), metabolome, metagenome, multi-omics, single-cell RNA sequencing

## Abstract

Alcohol-related liver disease (ALD) is a major cause of liver-related morbidity and mortality worldwide, yet the associations linking alcohol-induced gut microbial alterations to metabolic remodeling and hepatocyte dysfunction remain incompletely understood. Here, we applied an integrative multi-omics strategy combining untargeted fecal metabolomics, shotgun metagenomics, mouse liver bulk RNA sequencing, and reanalysis of publicly available human hepatic single-cell and bulk transcriptomic datasets to characterize alcohol exposure-associated gut–liver immunometabolic features. In a mouse model of acute ethanol-induced liver injury, fecal metabolomic and metagenomic profiling revealed marked alterations in microbial functional potential and fecal metabolic composition, identifying six convergent metabolic pathways across fecal multi-omics layers, including nucleotide metabolism, the pentose phosphate pathway, histidine metabolism, glycerophospholipid metabolism, glycine/serine/threonine metabolism, and the phosphotransferase system. Reanalysis of human ALD single-cell transcriptomes showed hepatocyte-enriched activity patterns for several corresponding pathways, suggesting potential pathway-level associations between fecal metabolic alterations and hepatic transcriptional responses. Integrative transcriptomic analysis further identified a ten-gene hepatocyte-associated signature, comprising LRG1, ORM1, ORM2, TAT, HP, FGB, FGG, ITIH3, NNMT, and AGT, which was associated with pathway activity and showed consistent upregulation across acute ethanol-induced liver injury and human ALD/AH transcriptomic datasets. In an external human cohort, this signature stratified patients into exploratory molecular subgroups with distinct metabolic pathway activities and clinical outcome distributions. Collectively, these findings provide a hypothesis-generating multi-omics framework for investigating alcohol-related liver injury and support further validation in chronic ethanol exposure models and functional studies.

## Introduction

Alcohol-related liver disease (ALD) remains a major cause of liver-related morbidity and mortality worldwide, encompassing a pathological continuum from simple steatosis and steatohepatitis to progressive fibrosis, cirrhosis, and hepatocellular carcinoma (1). Despite advances in understanding alcohol-induced oxidative stress, inflammation, and metabolic dysregulation, effective therapeutic options remain limited beyond alcohol abstinence. This unmet clinical need highlights the importance of delineating the inflammatory and immunometabolic programs that drive alcohol-related liver injury and may serve as actionable targets for therapeutic intervention (2).

Over the past decade, accumulating evidence has established the gut–liver axis as a central driver of ALD pathogenesis. Chronic alcohol consumption disrupts intestinal barrier integrity, reshapes gut microbial composition, and alters microbial functional outputs, thereby facilitating the translocation of microbial products and metabolites to the liver via the portal circulation (3). These gut-derived signals directly modulate hepatic metabolism, immune activation, and injury responses, indicating that alcohol-induced gut dysbiosis generates metabolically and immunologically active cues that promote inflammatory dysregulation in ALD (4). Importantly, emerging studies have demonstrated that targeting gut microbial communities or their metabolic activities can ameliorate alcohol-induced liver injury (5, 6). For example, dietary fiber supplementation has been shown to improve ALD phenotypes in mice by remodeling the gut microbiota and modulating hepatocyte metabolic pathways through bile acid–FXR–FGF15 signaling, underscoring the therapeutic potential of microbiota- and metabolite-centered interventions (7).

In parallel, advances in multi-omics profiling have revealed that ALD is characterized by extensive metabolic reprogramming, including perturbations in redox homeostasis, amino acid metabolism, lipid remodeling, and energy metabolism (8). These metabolic alterations converge predominantly on hepatocytes, which function as the principal metabolic hub of the liver and represent the first line of cellular response to gut-derived metabolites and toxins. Consistent with this concept, recent mechanistic studies have shown that hepatocyte-intrinsic metabolic regulators, such as NAD^+^-associated enzymes, play critical protective roles in limiting alcohol-induced lipid accumulation and oxidative stress, further reinforcing hepatic metabolic pathways as key determinants of disease severity (9).

Despite increasing recognition of gut–liver metabolic communication in ALD, two key knowledge gaps remain. First, the specific metabolic pathways that concurrently capture gut microbial functional shifts and fecal metabolic remodeling in response to alcohol exposure have not been systematically defined. Second, how these gut-derived metabolic signatures are translated into cell-type–specific hepatic inflammatory and transcriptional programs—particularly within hepatocytes—remains poorly understood. Addressing these gaps is essential for establishing mechanistic links between intestinal multi-omics alterations and hepatic injury, and for identifying biomarkers that enable stratification of ALD heterogeneity and inform precision therapeutic strategies.

In this study, we integrated untargeted fecal metabolomics with shotgun metagenomics to delineate alcohol-associated functional and metabolic alterations within the gut ecosystem and to identify convergent pathway signals consistently enriched across both datasets. We then leveraged publicly available single-cell transcriptomic data from ALD liver biopsies to map the activity of these shared pathways at the cellular level, with a particular focus on hepatocytes. By integrating hepatocyte-specific transcriptional signatures from single-cell RNA sequencing (scRNA-seq) with bulk liver RNA-seq profiles, we prioritized candidate genes associated with ALD pathogenesis. Finally, we validated the expression of these candidate genes in an independent mouse cohort and assessed their potential clinical relevance using an external ALD patient transcriptomic dataset, including unsupervised molecular subtyping. Collectively, this integrative multi-omics framework provides a systems-level view of gut-derived metabolic remodeling and hepatocyte-associated transcriptional reprogramming in ALD, and offers a resource for identifying disease-associated pathways, candidate biomarkers, and potential intervention targets along the gut–liver axis.

## Materials and methods

### Animals

All animal experiments were conducted in accordance with ethical guidelines for laboratory care and use. The study protocol was approved by the Institutional Animal Care and Use Committee (IACUC) of the South China University of Technology. Male C57BL/6 mice (6–8 weeks old) were obtained from Hunan SJA Laboratory Animal Co., Ltd. Mice were assigned to Control or acute ethanol groups using a randomization list generated before gavage. Samples and histological slides were coded during sample processing and image acquisition. Histological scoring and quantitative image evaluation were performed by investigators blinded to group allocation until primary measurements had been completed.

### Establishment of acute liver injury models

Male C57BL/6J mice were used to establish acute alcohol-induced liver injury models. For the acute alcohol-induced liver injury model, mice were fasted for 12 h prior to administration of a single dose of 50% (v/v) ethanol (6 g/kg) by oral gavage. At 24 h after ethanol administration, mice were anesthetized by inhalation of isoflurane at 3–4% for induction in oxygen and subsequently euthanized by gradual-fill carbon dioxide inhalation. Briefly, mice were placed in a 10-L euthanasia chamber without pre-charging, and 100% CO2 was introduced at a flow rate of 5 L/min, corresponding to 50% of the chamber volume per minute. CO2 flow was maintained for at least 1 min after respiratory arrest, and death was confirmed before liver tissues were promptly harvested for subsequent analyses.

### Transcriptome sequencing

Total RNA was extracted from liver tissue samples using TRIzol reagent (Invitrogen, CA, USA) according to the manufacturer’s protocol. RNA concentration and purity were assessed using a NanoDrop ND-1000 spectrophotometer, and RNA integrity was evaluated with an Agilent 2100 Bioanalyzer. Samples with RNA concentration > 50 ng/μL, total RNA > 1 μg, RNA integrity number (RIN) > 7.0, and OD260/280 > 1.8 were used for library preparation.

RNA sequencing libraries were constructed using standard protocols and sequenced on an Illumina NovaSeq 6000 platform (LC Bio Technology Co., Ltd., Hangzhou, China) using a paired-end 150 bp configuration. Raw sequencing reads were processed to remove adaptor sequences, low-quality reads, and reads containing ambiguous bases. Clean reads were aligned to the reference genome using standard alignment pipelines, and gene expression levels were quantified as fragments per kilobase of transcript per million mapped reads (FPKM) or transcripts per million (TPM), as indicated. The raw mouse liver bulk RNA-seq data generated in this study have been deposited in the Gene Expression Omnibus (GEO) database under accession number GSE331211.

Differential gene expression analysis between experimental groups was performed using established statistical methods implemented in R. Genes with an adjusted P value (false discovery rate, FDR) < 0.05 and |log_2_ fold change| > 1 were defined as differentially expressed genes (DEGs). Functional enrichment analyses were conducted using Gene Ontology (GO) and Kyoto Encyclopedia of Genes and Genomes (KEGG) databases to identify significantly enriched biological processes and pathways, with FDR < 0.05 considered statistically significant. In addition, gene set enrichment analysis (GSEA) was performed to assess coordinated expression changes across predefined gene sets without applying a fold-change threshold.

### Untargeted fecal metabolomics profiling and analysis

Untargeted fecal metabolomics was performed on snap-frozen fecal samples stored at −80 °C. Fecal samples from Control mice (n = 6) and acute ethanol-exposed mice (n = 6) were included. Metabolites were extracted using a cold methanol/acetonitrile/water mixture, followed by vortexing, sonication, and centrifugation. Supernatants were collected for analysis, and a pooled quality control (QC) sample was prepared by combining equal aliquots from all samples to monitor analytical stability.

Metabolomic profiling was conducted using ultra-high-performance liquid chromatography coupled with tandem mass spectrometry (UHPLC–MS/MS) in both positive and negative electrospray ionization modes on a high-resolution platform (LC-Bio Technology, Hangzhou, China). Raw data were processed for peak detection, retention time alignment, and signal normalization. Metabolite annotation was performed based on accurate mass, retention time, and MS/MS spectra using reference libraries and public databases, including the Human Metabolome Database (HMDB), METLIN, and MassBank.

Principal component analysis (PCA) was used to evaluate global metabolic variation and QC clustering. Differential metabolites between Control and acute ethanol-exposed mice were identified using combined multivariate and univariate analyses. Fold change, nominal P value, Benjamini–Hochberg-adjusted q value, and variable importance in projection (VIP) score were calculated for each metabolite. Metabolites with |log_2_ fold change| > 1, P < 0.05, and VIP > 1 were considered differentially altered, and q values were reported to indicate FDR-adjusted significance. Detailed metabolite annotation information, including ion type, metabolite ID, mass-to-charge ratio (MZ), retention time (RT), MS2 annotation, MS2 score, molecular formula, HMDB ID, KEGG ID, KEGG pathway annotation, sample-level intensity values, group means, fold changes, P values, q values, VIP scores, and significance labels, is provided in **Supplementary Table 2**. KEGG pathway enrichment and metabolite correlation analyses were performed to identify perturbed metabolic pathways and coordinated metabolite modules. All statistical analyses were performed using R-based pipelines.

### Shotgun metagenomic sequencing and analysis

Total microbial DNA was extracted from fecal samples using a commercial stool DNA extraction kit with bead-beating to ensure efficient lysis. DNA quantity and quality were assessed, and qualified samples were used for library preparation according to standard Illumina protocols.

Shotgun metagenomic sequencing was conducted on the Illumina NovaSeq 6000 platform using a paired-end 150 bp (PE150) strategy (LC Bio Technology, Hangzhou, China). Raw reads were filtered to remove adaptor sequences, low-quality reads, and host-derived contaminants. Clean reads were used for downstream taxonomic and functional profiling. Microbial taxonomic composition was determined across multiple taxonomic levels, and differential abundance analysis was performed between experimental groups. Linear discriminant analysis effect size (LEfSe) analysis was performed to identify microbial taxa discriminating using default parameters, and taxa with log-transformed LDA scores exceeding the predefined threshold were considered discriminative. Taxonomic profiles are provided in **Supplementary Table 3**.

For functional profiling, microbial genes were annotated against the KEGG database, and pathway abundance profiles were compared between groups to identify differentially enriched pathways. In addition, correlation analysis was conducted to assess structured associations among differentially abundant taxa. All analyses were performed using established computational pipelines and R-based tools.

### Integrated metagenomic–metabolomic analysis

Integrated metagenomic–metabolomic analysis was performed to assess pathway-level concordance between fecal microbial functional potential and fecal metabolite profiles after acute ethanol exposure. Paired metagenomic and metabolomic datasets obtained from the same fecal samples were used whenever available. Metagenomic functional profiles were annotated against the KEGG database, and metabolomic pathway enrichment was performed based on KEGG-mapped differential metabolites. Pathways identified in both omics layers were defined as shared pathways, and corresponding enrichment statistics, effect sizes, nominal P values, and Benjamini–Hochberg false discovery rate (FDR)-adjusted P values were reported.

To evaluate global cross-omics correspondence, Procrustes analysis was performed using KEGG pathway-level matrices derived from metagenomic and metabolomic data. Statistical significance was assessed by permutation testing, and the Procrustes statistic M² and permutation-based P value were reported. This analysis was interpreted as cross-omics co-variation rather than causal coupling.

Spearman rank correlation analysis was used to assess associations between differentially abundant microbial taxa and significantly altered fecal metabolites. Correlation coefficients, nominal P values, and FDR-adjusted P values were calculated. For microbiome compositional data, low-abundance features were filtered, and abundance profiles were transformed before correlation analysis. Interaction networks were constructed to visualize significant microbe–metabolite associations and pathway-level concordance.

### Dataset acquisition

Public bulk liver transcriptomic datasets GSE28619 and GSE94417 were integrated for external validation of the ten hepatocyte-associated candidate genes. For each dataset, normalized gene expression matrices and corresponding clinical annotation files were downloaded from the GEO database. Gene symbols were used for probe-to-gene mapping, and when multiple probes mapped to the same gene, the average expression value was used. Genes shared by both datasets were retained for downstream analysis. Expression values were log2-transformed when necessary and then merged according to common gene symbols.

To minimize dataset-specific technical variation, batch effects between GSE28619 and GSE94417 were corrected using the ComBat algorithm, with dataset origin defined as the batch variable. Group information was retained as the biological variable during batch correction. After batch correction, expression values of the ten hepatocyte-associated candidate genes, including LRG1, ORM1, ORM2, TAT, HP, FGB, FGG, ITIH3, NNMT, and AGT, were extracted and converted to z-scores for visualization and statistical comparison across Control, Alcoholic steatosis, Mild acute alcoholic hepatitis, Alcoholic hepatitis, and Severe acute alcoholic hepatitis groups. Kruskal–Wallis tests were used to compare gene expression across multiple groups.

### Single-cell RNA-seq data processing and analysis

Publicly available human liver single-cell RNA-seq data from GSE236382 were reanalyzed in this study. This dataset included liver biopsy samples from five patients with ALD. No healthy liver samples or non-ALD disease-control samples were available in this dataset. In addition, detailed clinical metadata, including fibrosis stage, inflammatory grade, MELD score, and other disease severity variables, were not uniformly available for all patients. Therefore, the single-cell analysis was used to define cell-type enrichment patterns within ALD liver tissues, but not to infer ALD-specific pathway activation or to perform patient-level severity-adjusted analysis. For initial quality control, genes detected in fewer than three cells were excluded, and cells with fewer than 200 detected genes or more than 20% mitochondrial transcripts were removed. The percentage of mitochondrial transcripts was calculated for each cell and used as an indicator of cell quality.

After quality control, each sample was normalized using log normalization with a scale factor of 10,000, and the top 5,000 highly variable genes were selected for integration. Sample integration was performed using an anchor-based integration strategy. The integrated expression matrix was scaled and subjected to principal component analysis (PCA) for dimensionality reduction. The first 30 principal components were used to construct the shared nearest-neighbor graph and perform unsupervised clustering at a resolution of 1. UMAP and t-SNE embeddings were generated using the first 20 principal components. Sample distribution across UMAP clusters was examined to assess whether clustering was primarily driven by sample origin.

Cell-types were annotated based on cluster-specific marker genes identified by differential expression analysis and canonical markers. Briefly, T cells were defined by IL7R, TRBC2, and CD2; cytotoxic T/NK cells by CCL5, NKG7, GNLY, GZMB, and KLRD1; myeloid/macrophage populations by LYZ, FCGR3A, IL1B, and C1QB, with antigen-presenting cells characterized by HLA-DRA and CD74. B cells were annotated using MS4A1, CD79A, IGHM, IGKC, and BANK1. Hepatic stellate cells (HSCs)/fibroblast-like cells were identified by DCN, IGFBP7, and TIMP1, together with activation markers TAGLN and ACTA2. Endothelial cells were defined by VWF, PECAM1, and SPARCL1, lymphatic endothelial cells by PROX1 and LYVE1, cholangiocytes by KRT18, KRT19, and EPCAM, and hepatocytes by ALB, APOE, APOC3, RBP4, and TTR. These major hepatic cell populations were visualized on UMAP.

To quantify host pathway activity at single-cell resolution, AUCell was applied to score host-interpretable KEGG pathways that overlapped with the integrated fecal metagenomic–metabolomic analysis, including nucleotide metabolism, the pentose phosphate pathway (PPP), histidine metabolism (HIS), glycerophospholipid metabolism, and glycine/serine/threonine metabolism (GST). Because the phosphotransferase system (PTS) is a bacterial-specific transport system and does not represent a host hepatocyte transcriptional pathway, it was excluded from the human single-cell AUCell analysis. AUCell scores were compared across annotated liver cell types, and feature plots were generated to visualize pathway activity in the UMAP embedding. These analyses were used to evaluate cell-type enrichment patterns of host metabolic gene programs within the ALD single-cell dataset, rather than to infer direct mechanistic continuity between microbial functional pathways and hepatocyte transcriptional activity. Candidate hepatocyte-associated genes were prioritized by intersecting significantly upregulated genes from mouse liver bulk RNA-seq after acute ethanol exposure with hepatocyte-associated marker genes derived from the reanalyzed human ALD single-cell transcriptomic dataset.

### Unsupervised consensus clustering and Chi-square test

Unsupervised consensus clustering was performed using the ConsensusClusterPlus package in R to identify molecular subtypes based on the expression profiles of the ten candidate genes in an independent cohort. Clustering robustness was assessed using a resampling-based strategy with 1,000 iterations and 80% subsampling of samples and features per iteration. The optimal number of clusters (k = 2–6) was determined based on consensus cumulative distribution function (CDF) curves and the relative change in the area under the CDF curve (ΔAUC).

Heatmaps of gene expression across molecular subtypes were generated using the pheatmap package in R. Differences in clinical or pathological category distributions between subtypes were evaluated using the Chi-square test. All statistical tests were two-sided, and P < 0.05 was considered statistically significant.

### Measurement of liver coefficient

At sacrifice, whole livers were excised, rinsed with ice-cold phosphate-buffered saline, blotted dry, and weighed immediately. Body weight was recorded on the same day. The liver coefficient was calculated as: (liver weight/body weight) × 100%.

### Hepatic oxidative stress assays

Liver tissues were homogenized in ice-cold lysis buffer and centrifuged to obtain supernatants. Superoxide dismutase (SOD) activity and malondialdehyde (MDA) levels were measured using commercial assay kits according to the manufacturers’ instructions. Total protein concentrations were determined by a BCA assay, and results were normalized to protein content (SOD: U/mg protein; MDA: nmol/mg protein).

### Cytokine quantification by ELISA

For *in vivo* assays, liver tissues were homogenized in ice-cold PBS (or kit buffer), centrifuged, and the supernatants were used for cytokine measurement. Concentrations of IL-10, TNF-α, and IL-6 in liver homogenates, were quantified using commercial enzyme-linked immunosorbent assay (ELISA) kits following the manufacturers’ protocols. Cytokine levels in tissue samples were normalized to total protein content.

### Serum ALT and AST measurement

Blood samples were collected at sacrifice, allowed to clot, and centrifuged to obtain serum. Serum alanine aminotransferase (ALT) and aspartate aminotransferase (AST) levels were measured using an automated biochemical analyzer or commercial colorimetric assay kits according to the manufacturers’ instructions, and reported in U/L.

### Histopathological analysis

At the end of the experiment, liver tissues were collected from each mouse and fixed in 4% paraformaldehyde, followed by paraffin embedding and sectioning at 4–5 μm thickness.

For histopathological evaluation, sections were stained with hematoxylin and eosin (H&E) according to the manufacturer’s instructions (Solarbio, China). Stained slides were examined under a light microscope (Nikon Ts2) to assess inflammatory cell infiltration, hepatocellular degeneration, and disruption of hepatic architecture and calculate the injury score (10). Representative images were captured for comparison among experimental groups.

### Real-time quantitative PCR analysis

Total RNA was extracted from mouse liver tissues using RNAiso Plus (Takara, Japan) according to the manufacturer’s instructions. Residual genomic DNA was removed using gDNA Eraser, and complementary DNA (cDNA) was synthesized using the PrimeScript RT reagent kit (Takara, Japan). RT-qPCR was performed using a SYBR Green qPCR kit (AG, China) on a LightCycler 96 system (Roche Diagnostics). Each 10 μL reaction contained 1 μL cDNA template, 5 μL SYBR Green Master Mix, 0.4 μL forward primer (10 μM), 0.4 μL reverse primer (10 μM), and 3.2 μL nuclease-free water. All reactions were performed with biological replicates from control mice and acute ethanol-exposed mice. Primer specificity was confirmed by melting curve analysis, and amplification efficiency was evaluated to ensure reliable quantification. β-actin was used as the internal reference gene because its expression remained stable across control and acute ethanol-exposed liver samples under the experimental conditions. Relative gene expression was calculated using the 2^−ΔΔCt method, where ΔCt = Ct_target − Ct_β-actin and ΔΔCt = ΔCt_ethanol − ΔCt_control. Primer sequences are listed in **Supplementary Table 4**.

### Statistical analysis

Quantitative data are presented as mean ± SEM unless otherwise indicated. For newly generated mouse experiments, each group contained six biological replicates, including Control mice (n = 6) and acute ethanol-exposed mice (n = 6). Continuous variables were inspected for distributional pattern and variance consistency before group comparison. Comparisons between two groups were performed using Student’s t-test for approximately normally distributed data with acceptable variance homogeneity, or the Wilcoxon rank-sum test when distributional assumptions were not met or when a non-parametric comparison was more appropriate. Categorical variables were analyzed using the Chi-square test or Fisher’s exact test. All reported n values represent biological replicates unless otherwise specified.

## Results

### Acute ethanol exposure alters fecal metabolomic profiles in mice

**Figure 1:**
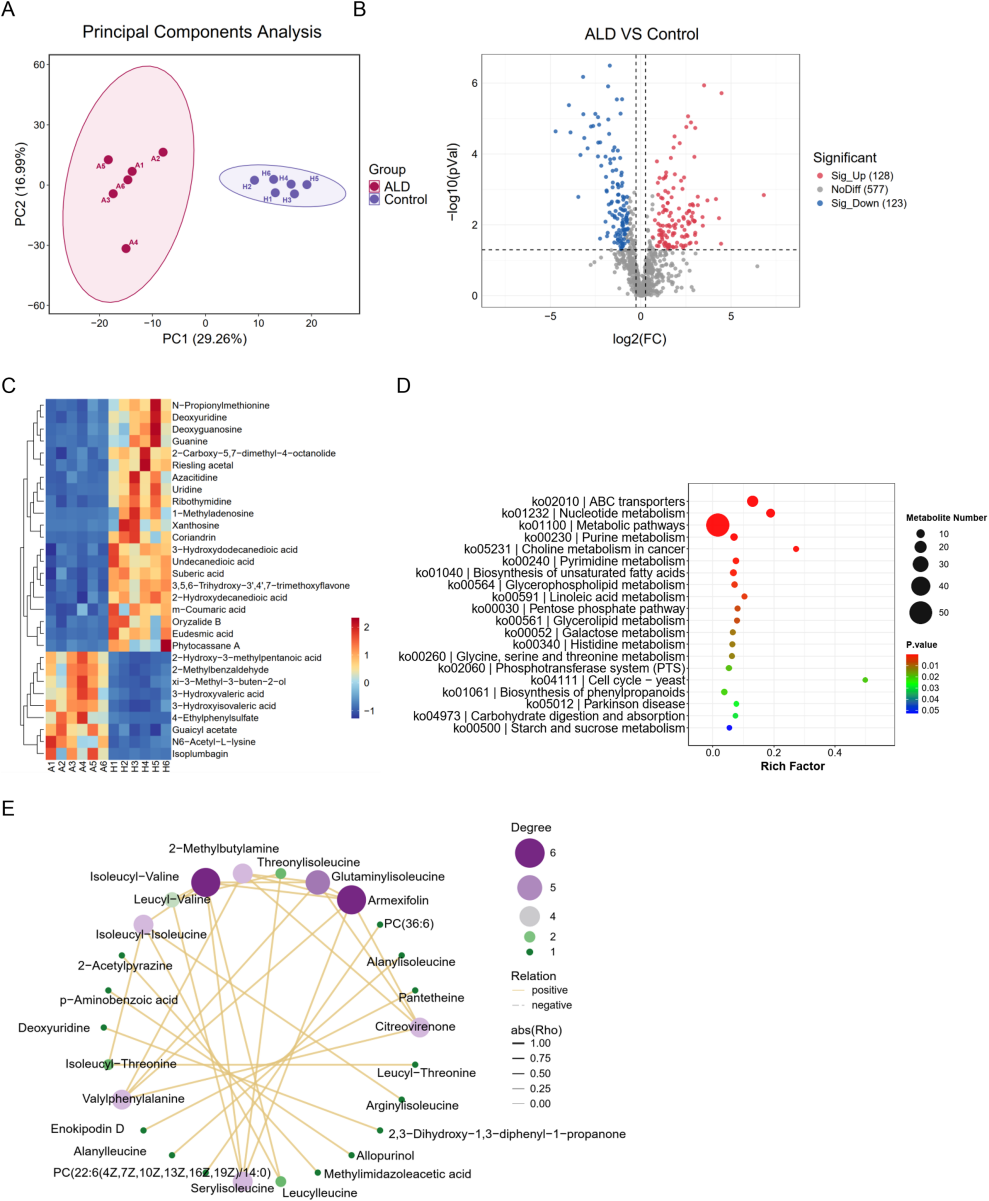
Acute ethanol exposure reshapes the fecal metabolomic landscape in mice. **(A)** Principal component analysis (PCA) of untargeted fecal metabolomic profiles from control and acute ethanol-exposed mice. **(B)** Differential metabolite analysis of fecal samples between control and acute ethanol-exposed mice. **(C)** Heatmap of differentially abundant fecal metabolites across samples. **(D)** KEGG pathway enrichment analysis of significantly altered metabolites. **(E)** Correlation network depicting structured associations among significantly altered metabolites.

To characterize alcohol-induced alterations in the gut metabolic environment and identify metabolic cues relevant to downstream immune dysregulation, untargeted fecal metabolomic profiling was performed in ALD and control mice. PCA revealed a clear separation between ALD and control samples along the first two principal components, which accounted for 29.26% and 16.99% of the total variance, respectively (**Figure 1A**), indicating a pronounced alcohol-driven remodeling of the fecal metabolic landscape.

Consistent with this global shift, differential metabolite analysis identified 251 significantly altered metabolites between the two groups (|log_2_ fold change| > 1, P < 0.05), comprising 128 upregulated and 123 downregulated metabolites in acute ethanol-exposed mice (**Figure 1B**), reflecting extensive metabolic remodeling following alcohol exposure. Hierarchical clustering based on these differential metabolites further demonstrated distinct and internally coherent metabolomic signatures for ALD and control mice (**Figure 1C**), reinforcing the reproducibility and biological relevance of the alcohol-associated metabolic alterations.

To delineate the functional implications of these metabolic alterations, KEGG pathway enrichment analysis was performed. Significantly enriched pathways included nucleotide metabolism, purine and pyrimidine metabolism, the PPP, HIS, GST, glycerophospholipid metabolism, and the PTS (**Figure 1D**). These findings indicate that acute ethanol exposure is associated with fecal metabolic alterations involving pathways related to redox regulation, amino acid metabolism, and lipid remodeling.

In addition, pairwise correlation analysis of alcohol-associated metabolites revealed structured networks of strong positive and negative associations (**Figure 1E**), indicating coordinated metabolic remodeling rather than isolated changes in individual metabolites. Given the established roles of these pathways in redox regulation, membrane remodeling, and inflammatory signaling, these alterations likely define a gut metabolic environment permissive to downstream immune and inflammatory perturbations. Collectively, these findings suggest that acute ethanol exposure is associated with coordinated fecal metabolic alterations, which may provide candidate metabolic features relevant to alcohol-related liver injury.

### Acute ethanol exposure is associated with altered gut microbial composition and functional potential in mice

**Figure 2:**
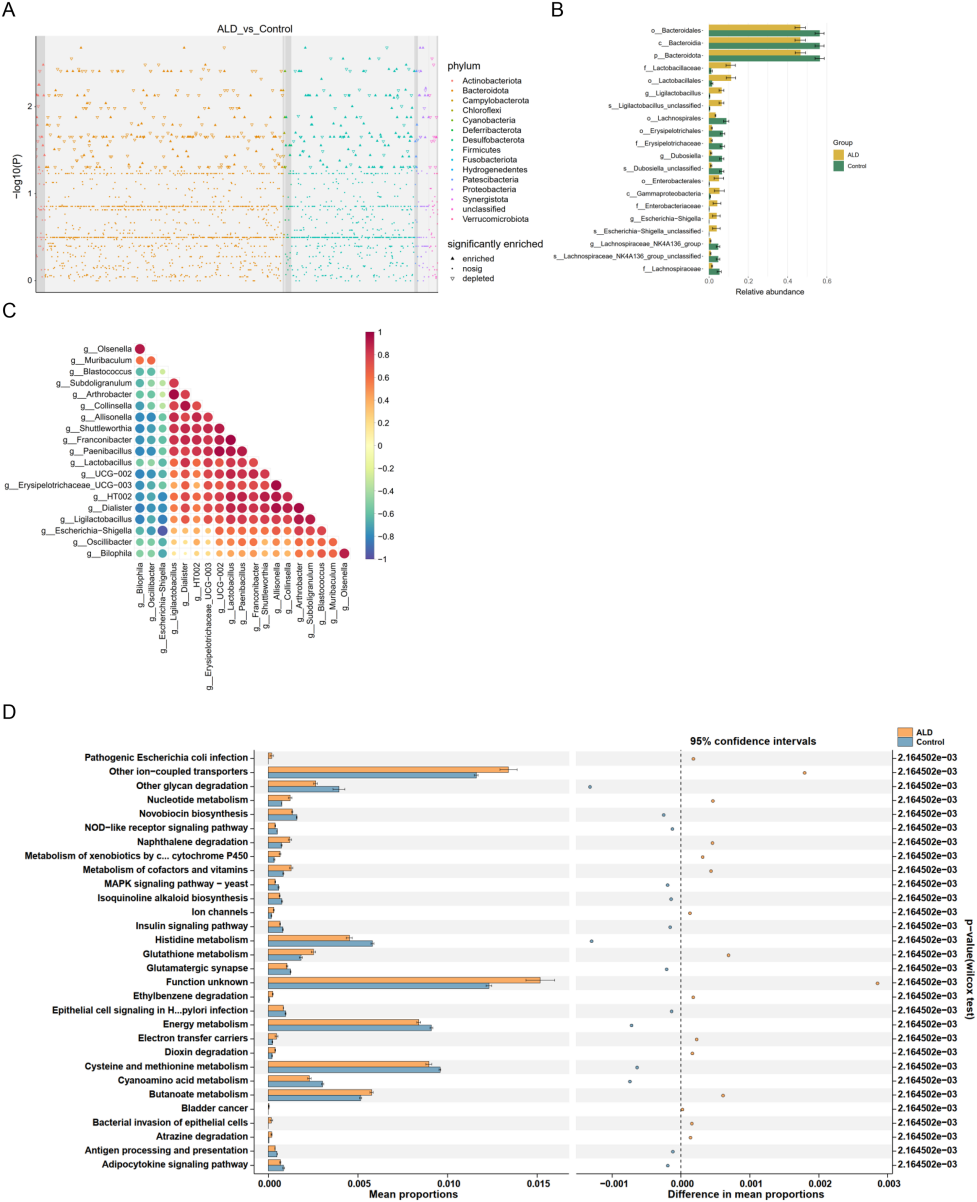
Alcohol exposure reshapes gut microbial composition and functional potential. **(A)** Manhattan plot depicting differentially abundant intestinal microbial species identified by shotgun metagenomic sequencing. **(B)** Linear discriminant analysis effect size (LEfSe) identifying microbial taxa discriminative of each experimental group. **(C)** Correlation matrix illustrating structured associations among differentially abundant microbial taxa. **(D)** Comparison of KEGG functional pathway profiles inferred from metagenomic annotations between groups.

To delineate microbial alterations linked to downstream immunometabolic dysregulation, we performed shotgun metagenomic sequencing of fecal samples from ALD and control mice. Differential abundance analysis revealed significant changes in microbial taxa between the two groups, with multiple microbial taxa significantly enriched or depleted in acute ethanol-exposed mice relative to controls (**Figure 2A**). These changes spanned several major bacterial phyla, indicating broad ecosystem-level restructuring of the gut microbiome following alcohol exposure.

To identify the microbial features most discriminative of alcohol exposure, we applied linear discriminant analysis effect size (LEfSe). This analysis highlighted distinct sets of taxa that were differentially enriched in ALD versus control mice, defining condition-specific microbial signatures (**Figure 2B**). These findings indicate that alcohol exposure not only reshapes global microbial community composition but also selectively enriches microbial lineages adapted to the alcohol-injured intestinal niche.

Correlation analysis among differentially abundant taxa further revealed structured association patterns, including clusters of positively and negatively correlated microbes (**Supplementary Table 5**; **Figure 2C**). This pattern suggests that alcohol exposure reshapes not only the presence of individual taxa but also the cooperative and competitive relationships within the microbial community, reflecting coordinated reorganization of gut microbial ecology.

At the functional level, metagenomic annotation-based profiling revealed significant alterations in multiple KEGG pathways between ALD and control mice (**Figure 2D**). These pathways were predominantly involved in nucleotide metabolism, HIS, energy metabolism, amino acid metabolism, and transport systems. Collectively, these results indicate that alcohol-induced dysbiosis remodels the functional output of the gut microbiome, thereby reshaping the repertoire of microbiome-derived signals that may influence hepatic immune homeostasis and inflammatory responses.

### Integrated fecal metagenomic and metabolomic analyses identify pathway-level concordance after acute ethanol exposure

**Figure 3:**
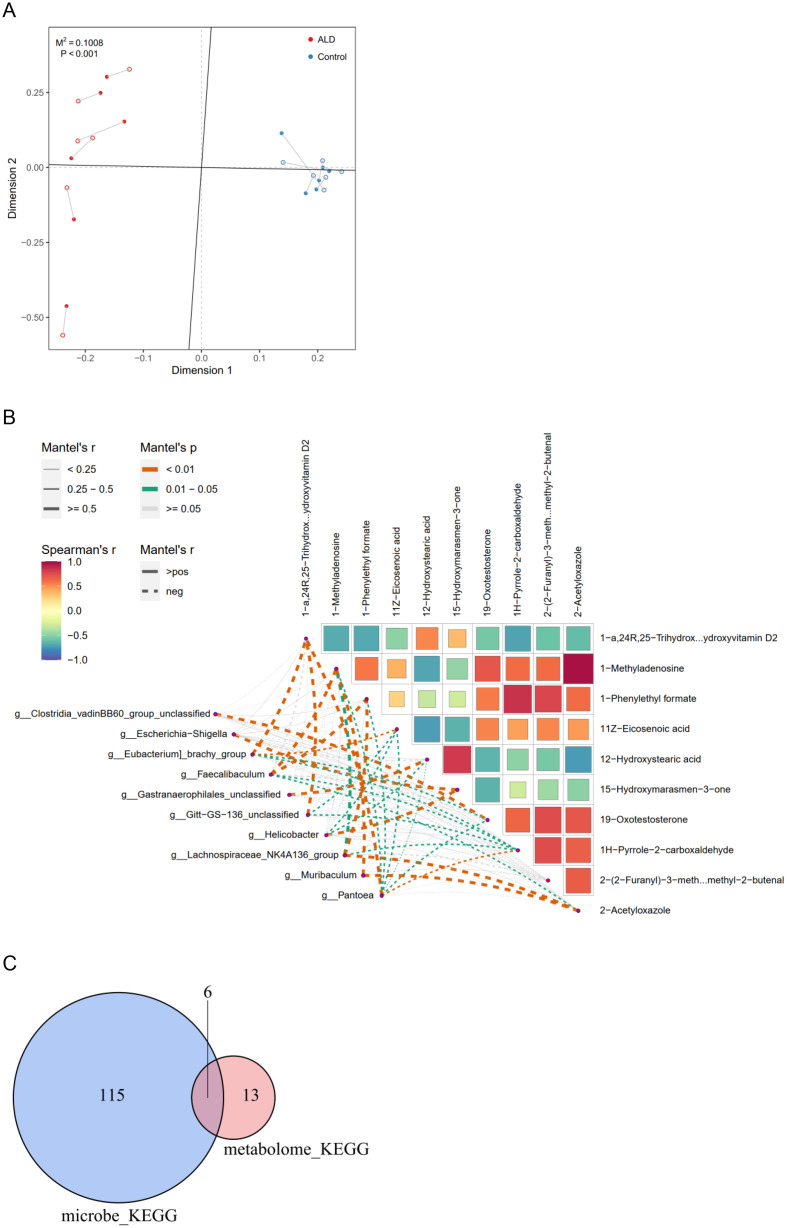
Integrated metagenomic and metabolomic analyses reveal pathway-level concordance after acute ethanol exposure. **(A)** Procrustes analysis based on KEGG pathway profiles derived from metagenomic and metabolomic datasets. **(B)** Heatmap showing correlations between differentially abundant microbial taxa and significantly altered fecal metabolites. **(C)** Venn diagram illustrating shared KEGG pathways identified by both metagenomic and metabolomic analyses.

To evaluate cross-omics concordance between fecal microbial functional potential and fecal metabolic alterations after acute ethanol exposure, we performed an integrated analysis of metagenomic and metabolomic datasets. Procrustes analysis based on KEGG pathway profiles showed a statistically significant global correspondence between the two omics layers, indicating co-variation between microbial functional potential and fecal metabolite composition (**Supplementary Table 6**; **Figure 3A**). However, this result should be interpreted as cross-omics correspondence rather than evidence of causal microbial–metabolic coupling.

Correlation analysis was further conducted between differentially abundant microbial taxa and altered fecal metabolites. This analysis identified structured patterns of positive and negative associations, with several bacterial genera statistically correlated with specific metabolites (**Supplementary Table 7**; **Figure 3B**). These findings suggest coordinated variation between microbial taxa and fecal metabolites after acute ethanol exposure, but do not establish that microbial changes directly caused the observed metabolite alterations.

At the pathway level, KEGG enrichment profiles from metagenomic and metabolomic analyses were systematically compared. Venn diagram analysis identified six shared pathways, including nucleotide metabolism, the PPP, HIS, glycerophospholipid metabolism, GST, and the PTS (**Supplementary Table 8**, **Figure 3C**). These pathways are related to redox homeostasis, amino acid metabolism, membrane remodeling, and microbial substrate utilization.

### Single-cell liver transcriptomic framework for immunometabolic analysis in ALD

**Figure 4:**
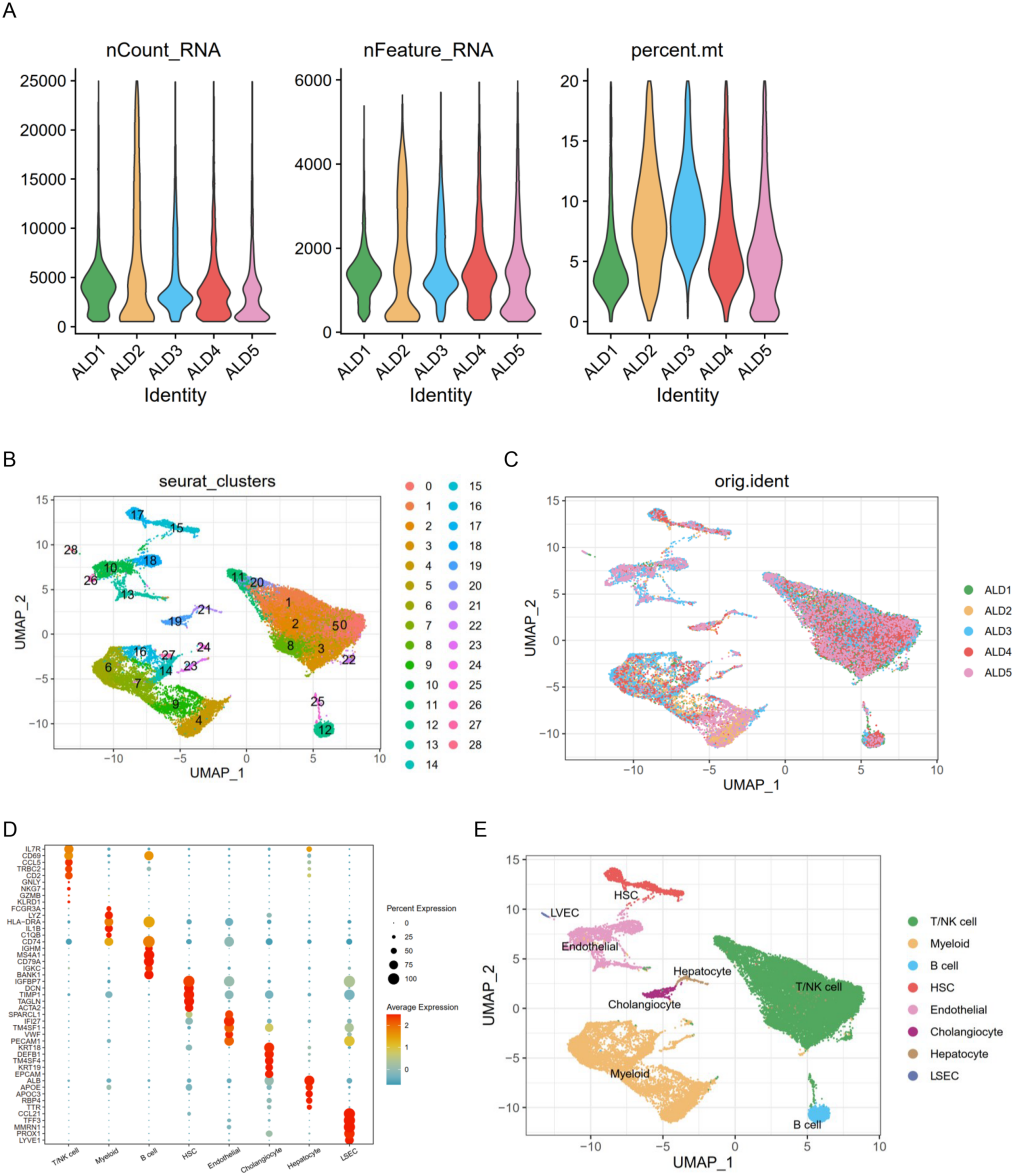
Quality control, clustering, and cell-type annotation of the reanalyzed human ALD liver scRNA-seq dataset. **(A)** Quality control metrics of single-cell RNA sequencing data, including nFeature_RNA, nCount_RNA, and mitochondrial gene percentage. **(B)** UMAP projection showing unsupervised clustering based on transcriptional profiles. **(C)** UMAP projection colored by sample origin. **(D)** Bubble heatmap showing canonical marker gene expression used for cell-type annotation across clusters. **(E)** UMAP projection colored by annotated cell types.

To delineate cell-type–specific transcriptional landscapes in ALD and provide a foundation for downstream immunometabolic analysis, we performed scRNA-seq on liver tissues. Following quality control filtering based on gene counts, RNA content, and mitochondrial gene proportion, high-quality cells were retained for downstream analyses (**Figure 4A**). The comparable distributions of key quality metrics (nCount_RNA, nFeature_RNA, and mitochondrial gene percentage) across samples indicate robust data quality and minimal technical bias.

Unsupervised clustering followed by UMAP dimensionality reduction identified multiple transcriptionally distinct cell populations that were well separated in low-dimensional space (**Figure 4B**). Cells colored by sample origin showed substantial inter-sample overlap within clusters (**Figure 4C**), indicating that the observed cellular organization was not driven by batch effects.

To assign biological identities, cluster-specific marker genes were identified and cross-referenced with canonical markers. Based on characteristic transcriptional signatures, major liver cell populations were annotated, including T/NK cells, myeloid cells, B cells, hepatic stellate cells (HSCs), endothelial cells, cholangiocytes, hepatocytes, and liver sinusoidal endothelial cells (LSECs) (**Figure 4D**). These cell types formed discrete and coherent clusters in UMAP space (**Figure 4E**), consistent with known liver cellular architecture. Collectively, these results establish a high-resolution single-cell transcriptional atlas of ALD liver tissue and provide the cellular basis for subsequent analyses of pathway activity and cell type–specific immunometabolic responses.

### Single-cell pathway activity scoring identifies hepatocyte-enriched activity of host metabolic pathways

To characterize cell type–associated host metabolic pathway activity in human ALD liver tissue (11), we applied AUCell to evaluate host-interpretable pathways corresponding to the integrated fecal metagenomic–metabolomic analysis, including nucleotide metabolism, the PPP, histidine metabolism (HIS), glycerophospholipid metabolism, and GST. The bacterial PTS was excluded from the human single-cell AUCell analysis because it represents a microbial transport system rather than a host transcriptional pathway.

**Figure 5:**
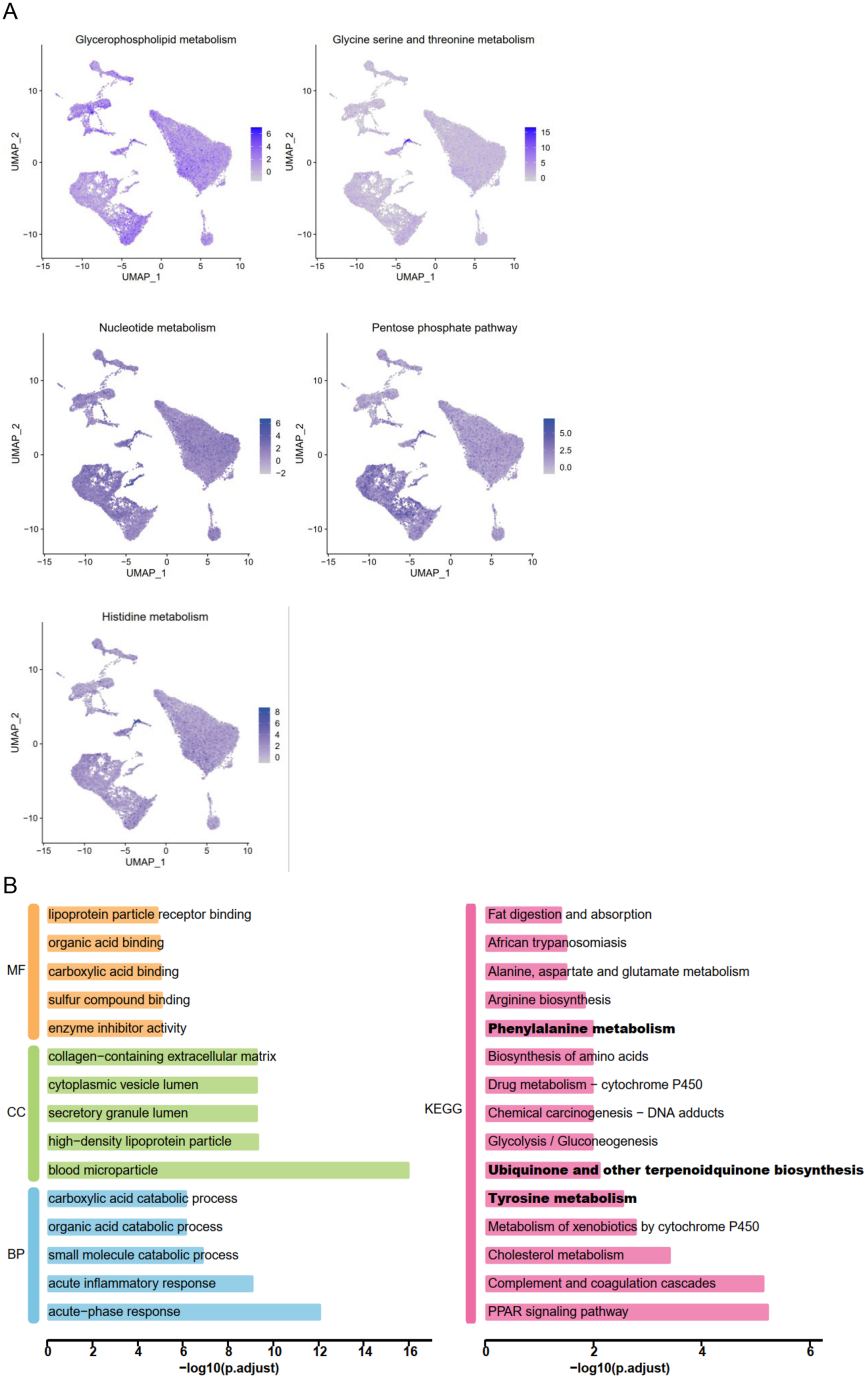
Hepatocyte-enriched metabolic activation and functional specialization in ALD liver. **(A)** AUCell-based scoring of shared KEGG pathway activities across annotated liver cell populations. **(B)** GO and KEGG enrichment analyses of cell-type specific marker genes derived from liver single-cell transcriptomes.

Across annotated liver cell populations, PPP, HIS, and GST showed relatively higher AUCell activity in hepatocytes than in other major cell types, indicating that these host metabolic gene programs were preferentially enriched in hepatocytes within the analyzed ALD single-cell dataset (**Figure 5A**). However, microbial KEGG pathway annotations and hepatocyte AUCell scores represent distinct biological layers. Therefore, these findings should be interpreted as cell-type localization of host metabolic programs rather than evidence of direct microbial-to-hepatocyte pathway continuity.

In addition, because the GSE236382 single-cell dataset lacks healthy liver or non-ALD disease-control samples and does not provide complete fibrosis stage, inflammatory grade, MELD score, or other disease-severity metadata for all patients, the observed hepatocyte-enriched pathway activity cannot be interpreted as ALD-specific activation. Instead, these results provide cellular context for host metabolic pathways within the available ALD liver samples.

To further characterize hepatocyte-associated transcriptional features, we performed functional enrichment analysis based on hepatocyte-marker genes (**Figure 5B**). GO analysis showed enrichment of terms related to lipid transport, organic acid metabolism, vesicular components, secretory activity, acute inflammatory response, and acute-phase response. KEGG analysis further highlighted pathways associated with phenylalanine and tyrosine metabolism, ubiquinone and terpenoid–quinone biosynthesis, glycolysis/gluconeogenesis, cholesterol metabolism, cytochrome P450-mediated xenobiotic metabolism, complement and coagulation cascades, and PPAR signaling.

### Transcriptomic profiling reveals coordinated metabolic and inflammatory transcriptional reprogramming in ALD liver

**Figure 6:**
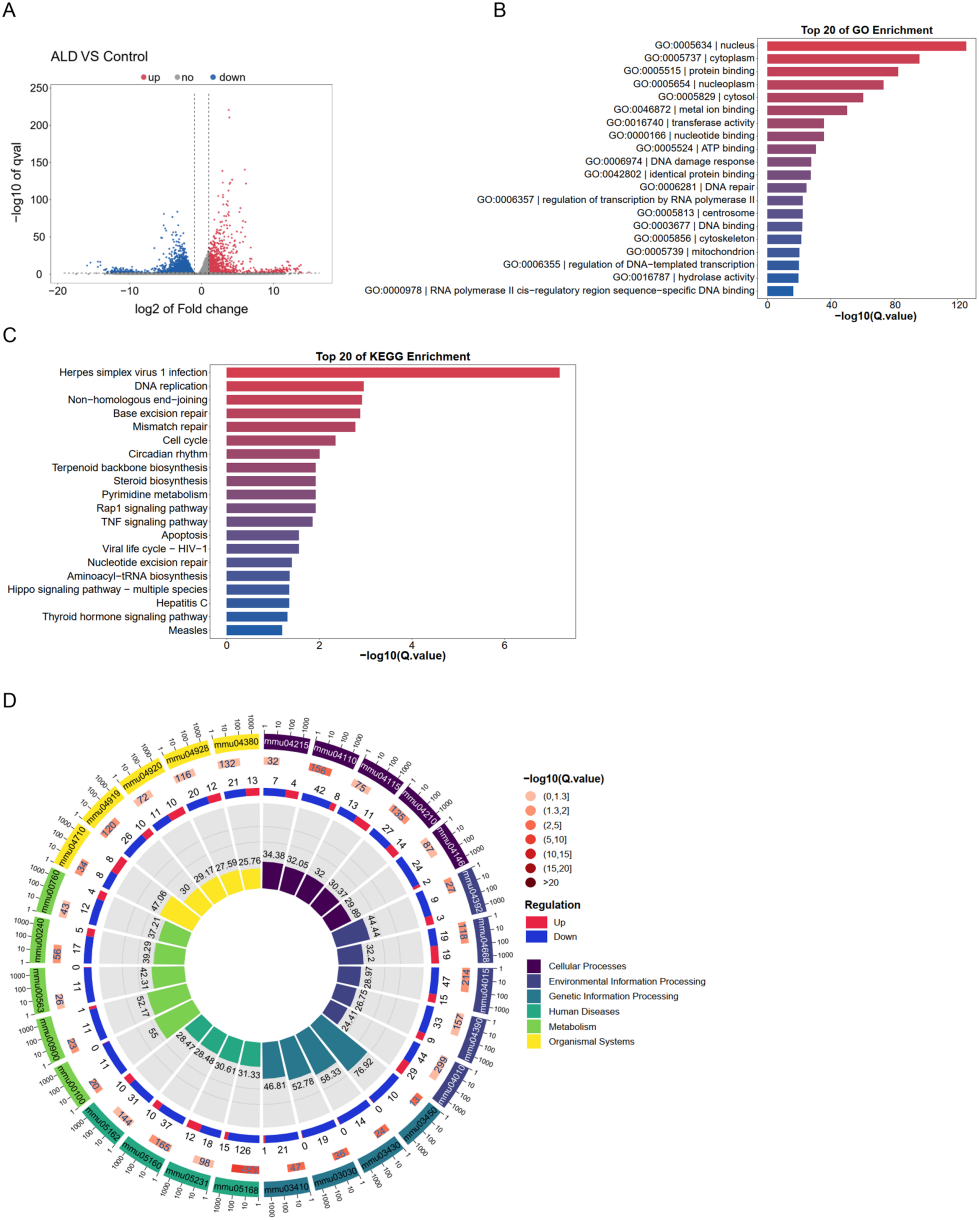
Bulk transcriptomic profiling reveals widespread transcriptional alterations in acute ethanol-exposed mouse liver tissue. **(A)** Volcano plot showing differential gene expression in liver tissues from control and alcohol-exposed mice. **(B)** GO enrichment analysis of differentially expressed genes. **(C)** KEGG pathway enrichment analysis of differentially expressed genes. **(D)** Circular KEGG enrichment plot summarizing globally enriched pathways and the directionality of gene regulation within each pathway.

To characterize transcriptional alterations associated with ALD, bulk RNA sequencing was performed on liver tissues from ALD and control mice. Differential expression analysis identified 5,138 significantly dysregulated genes in ALD livers, including 1,133 upregulated and 4,005 downregulated genes (adjusted P < 0.05; |log_2_ fold change| > threshold) (**Figure 6A**), indicating widespread transcriptional reprogramming induced by alcohol exposure.

To interpret the functional implications of these changes, GO enrichment analysis was conducted. Differentially expressed genes were predominantly enriched in fundamental cellular components and molecular functions, including nucleus, cytoplasm, nucleoplasm, cytosol, protein binding, and ATP binding (**Figure 6B**), suggesting broad perturbations in core cellular organization and biochemical activities.

KEGG pathway analysis further revealed that transcriptional alterations were non-random and preferentially concentrated in biologically coherent pathways related to genome stability, cell cycle control, metabolic regulation, and inflammatory signaling. Prominently enriched pathways included DNA replication, non-homologous end joining, base excision repair, mismatch repair, cell cycle, circadian rhythm, terpenoid backbone biosynthesis, steroid biosynthesis, pyrimidine metabolism, and the TNF signaling pathway (**Figure 6C**). These findings highlight the coordinated disruption of DNA repair, proliferative control, metabolic regulation, and inflammatory responses in ALD livers.

To integrate these alterations at a systems level, we generated a circular KEGG enrichment plot to visualize the global landscape of pathway-level changes and the directionality of gene regulation within each pathway (**Figure 6D**). This integrative view reveals coordinated yet heterogeneous liver-wide transcriptional remodeling across metabolic, stress-response, and inflammatory processes in ALD. Collectively, these results demonstrate that alcohol exposure drives extensive liver transcriptional reprogramming characterized by tightly coupled metabolic and inflammatory alterations, thereby providing tissue-level support for the hepatocyte-associated immunometabolic programs identified in single-cell analyses.

### Integrated transcriptomics identifies ten hepatocyte-associated genes linked to immunometabolic reprogramming

**Figure 7:**
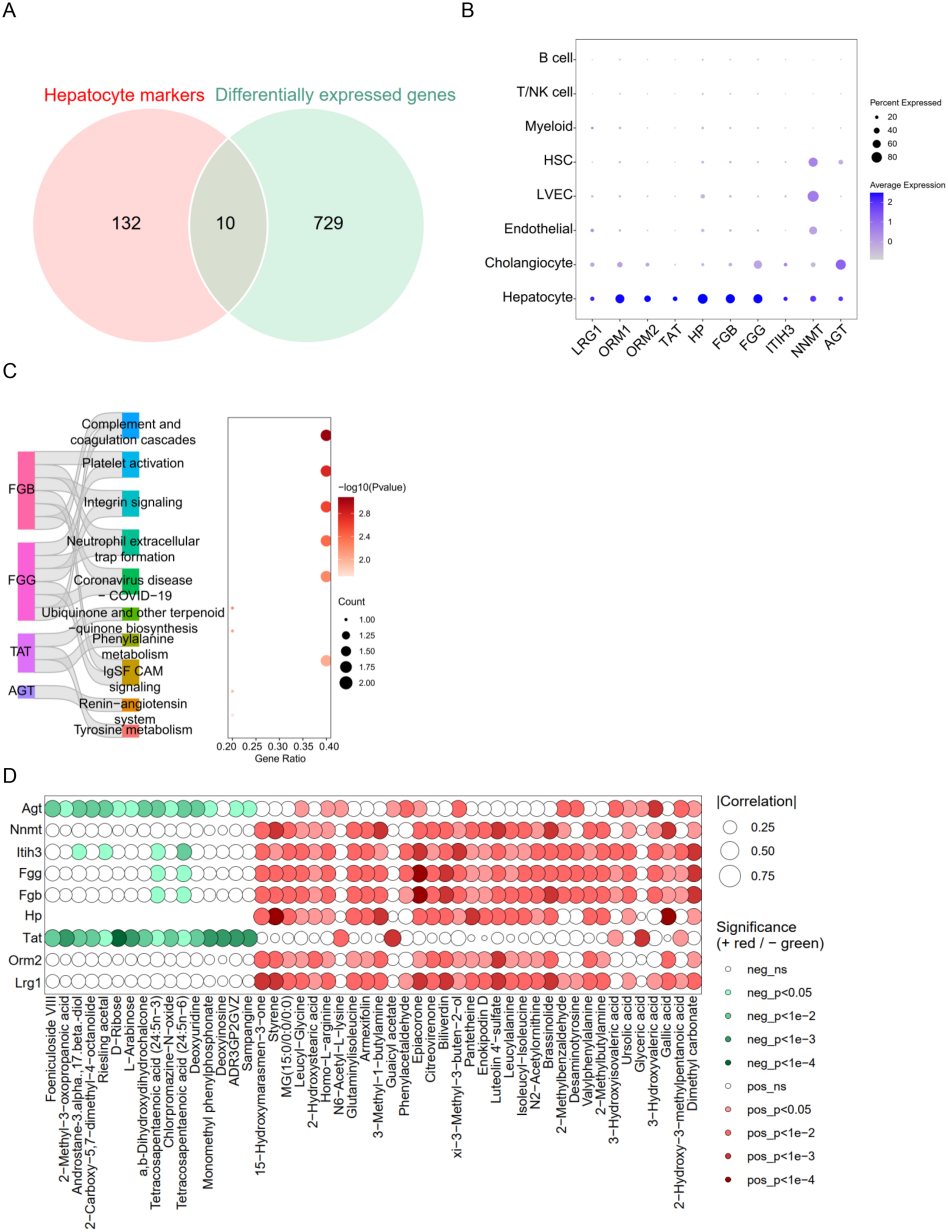
Integrative transcriptomic analyses identify hepatocyte-associated candidate genes in alcohol-related liver injury. **(A)** Schematic illustration of the intersection between genes upregulated in mouse liver bulk RNA sequencing and hepatocyte-associated marker genes derived from the reanalyzed human liver scRNA-seq dataset. **(B)** Dot plot showing the expression distribution of the ten candidate genes across major liver cell populations. **(C)** KEGG pathway enrichment analysis of the identified hepatocyte-associated candidate genes. **(D)** Correlation analysis between candidate gene expression levels and significantly altered fecal metabolites.

To define hepatocyte-associated genes consistently upregulated in alcohol-related liver injury, we integrated bulk liver transcriptomic data with hepatocyte marker genes derived from scRNA-seq analysis. Significantly upregulated genes in acute ethanol-induced liver injury livers (|log_2_ fold change| > 1) were intersected with hepatocyte-associated marker genes from the single-cell transcriptomic atlas to prioritize transcripts that were both hepatocyte-enriched and disease-associated. This integrative analysis identified ten overlapping genes (**Figure 7A**; **Supplementary Figure 1**): LRG1, ORM1, ORM2, TAT, HP, FGB, FGG, ITIH3, NNMT, and AGT. To further examine their cellular distribution, we visualized the expression of these genes across major liver cell populations using scRNA-seq data. As shown in the dot plot (**Figure 7B**), most of these genes were predominantly enriched in hepatocytes, supporting their designation as hepatocyte-associated genes, although selected genes such as NNMT and AGT also showed expression in limited non-parenchymal populations. Pathway enrichment analysis of this gene set revealed significant association with complement and coagulation cascades, platelet activation, integrin-mediated signaling, phenylalanine and tyrosine metabolism, ubiquinone and other terpenoid–quinone biosynthesis, and the renin–angiotensin system (**Figure 7C**), linking hepatocyte-associated transcriptional changes to inflammatory, metabolic, and vascular-related processes in alcohol-related liver injury.

To further bridge hepatocyte transcriptional changes with gut metabolic alterations, we conducted correlation analysis between the expression levels of the ten hepatocyte-enriched genes and differentially abundant fecal metabolites. This analysis revealed distinct patterns of positive and negative associations (**Figure 7D**), indicating that individual genes are selectively coupled to specific metabolic signatures rather than uniformly associated with global metabolomic shifts. Collectively, these results define a hepatocyte-associated gene module that links single-cell-resolved transcriptional programs with bulk liver remodeling and gut–liver immunometabolic crosstalk, and highlight these genes as potential molecular readouts of hepatocyte inflammatory and metabolic adaptation during alcohol-related liver injury.

### Unsupervised consensus clustering based on ten candidate genes identifies two clinically distinct subtypes in an independent ALD cohort

**Figure 8:**
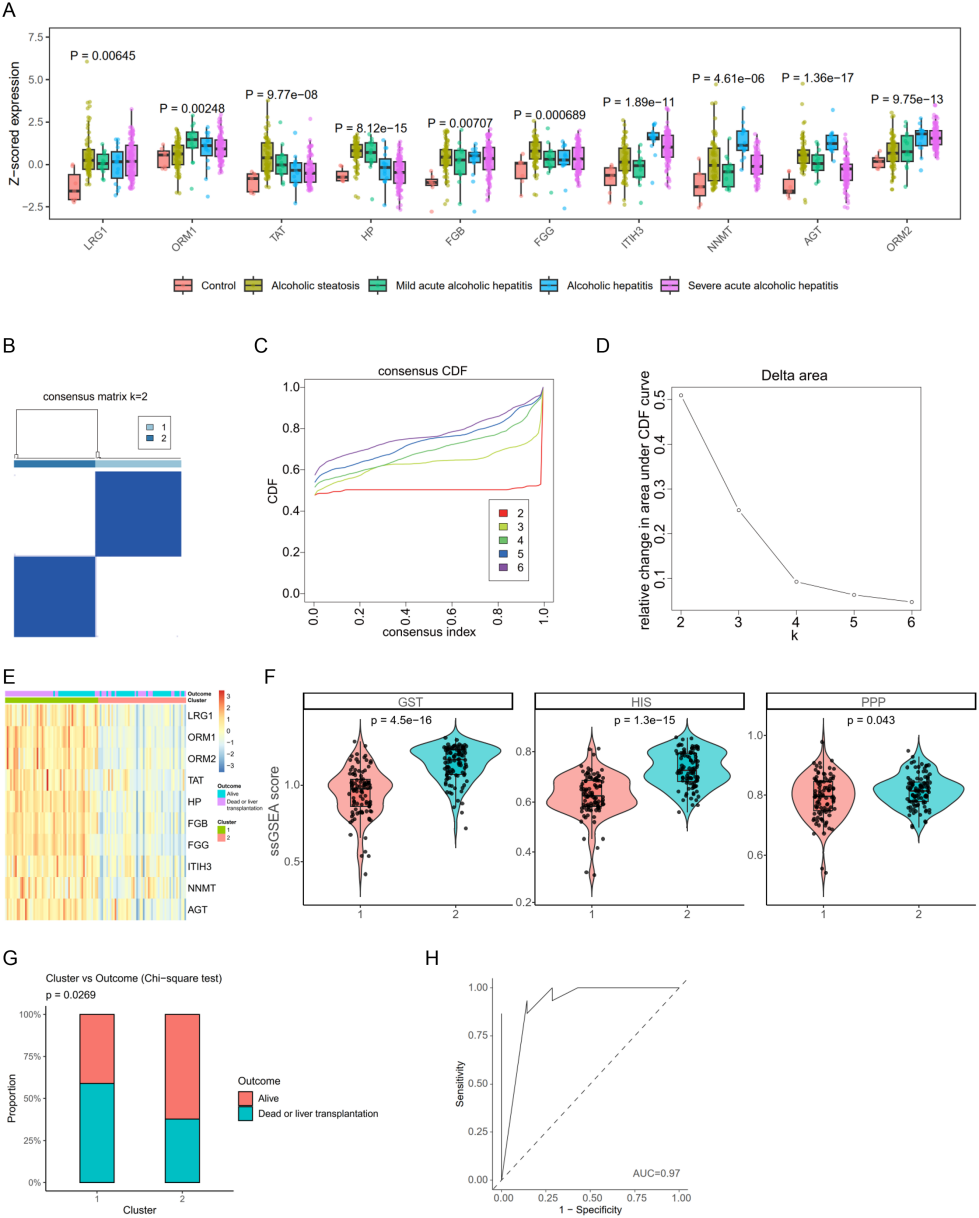
Molecular subtyping and pathway associations in an independent ALD cohort. **(A)** Expression patterns of the ten hepatocyte-associated candidate genes across control and alcohol-related liver injury/AH groups. **(B)** Consensus clustering matrix. **(C)** Consensus cumulative distribution function (CDF) curves for k = 2–6. **(D)** Relative change in the area under the CDF curve. **(E)** Heatmap of the ten candidate genes with subgroup and clinical outcome annotations. **(F)** Violin plots of GST, HIS, and PPP ssGSEA scores across exploratory molecular subgroups. GST, glycine/serine/threonine metabolism; HIS, histidine metabolism; PPP, pentose phosphate pathway. **(G)** Distribution of clinical outcome categories across exploratory molecular subgroups. **(H)** ROC curve of the ten-gene signature.

We first examined the expression of the ten hepatocyte-associated candidate genes across different alcohol-related liver injury stages in GSE94417 and GSE28619. As shown in **Figure 8A**, LRG1, ORM1, ORM2, TAT, HP, FGB, FGG, ITIH3, NNMT, and AGT were significantly differentially expressed among Control, Alcoholic steatosis, Mild acute alcoholic hepatitis, Alcoholic hepatitis, and Severe acute alcoholic hepatitis groups, with generally higher expression in alcohol-related disease groups than in controls. We then evaluated whether these genes could capture molecular heterogeneity among patients with ALD by performing unsupervised consensus clustering in the independent GSE94417 cohort. Clustering stability was assessed across k = 2–6 using consensus cumulative distribution function (CDF) curves and relative changes in the area under the CDF curve, which supported k = 2 as the optimal solution (**Figures 8B–D**). Heatmap visualization further showed distinct subtype-specific expression patterns of the ten-gene signature (**Figure 8E**), suggesting that this gene set may reflect transcriptional heterogeneity across ALD patients.

To explore the functional basis of this stratification, we compared pathway activities between the two subtypes using single-sample gene set enrichment analysis (ssGSEA). This analysis revealed significant differences in three metabolism-related pathways—GST, HIS, and the PPP—indicating that the identified molecular subtypes are characterized by distinct hepatocyte-associated immunometabolic states (**Figure 8F**).

To explore the potential clinical relevance of the ten-gene hepatocyte-associated signature, we next examined its association with patient outcome categories and its ability to distinguish disease samples from controls. Chi-square analysis revealed a significant association between cluster assignment and clinical outcome categories (P = 0.0269; **Figure 8G**), suggesting that the transcriptomically defined subgroups may reflect differences in disease-related molecular states. ROC analysis showed that this signature exhibited discriminatory ability for separating alcohol-related liver injury/AH samples from controls in the merged GSE28619 and GSE94417 datasets (AUC = 0.97, **Figure 8H**).

### Candidate hepatocyte-associated genes are upregulated in acute ethanol-induced liver injury

**Figure 9:**
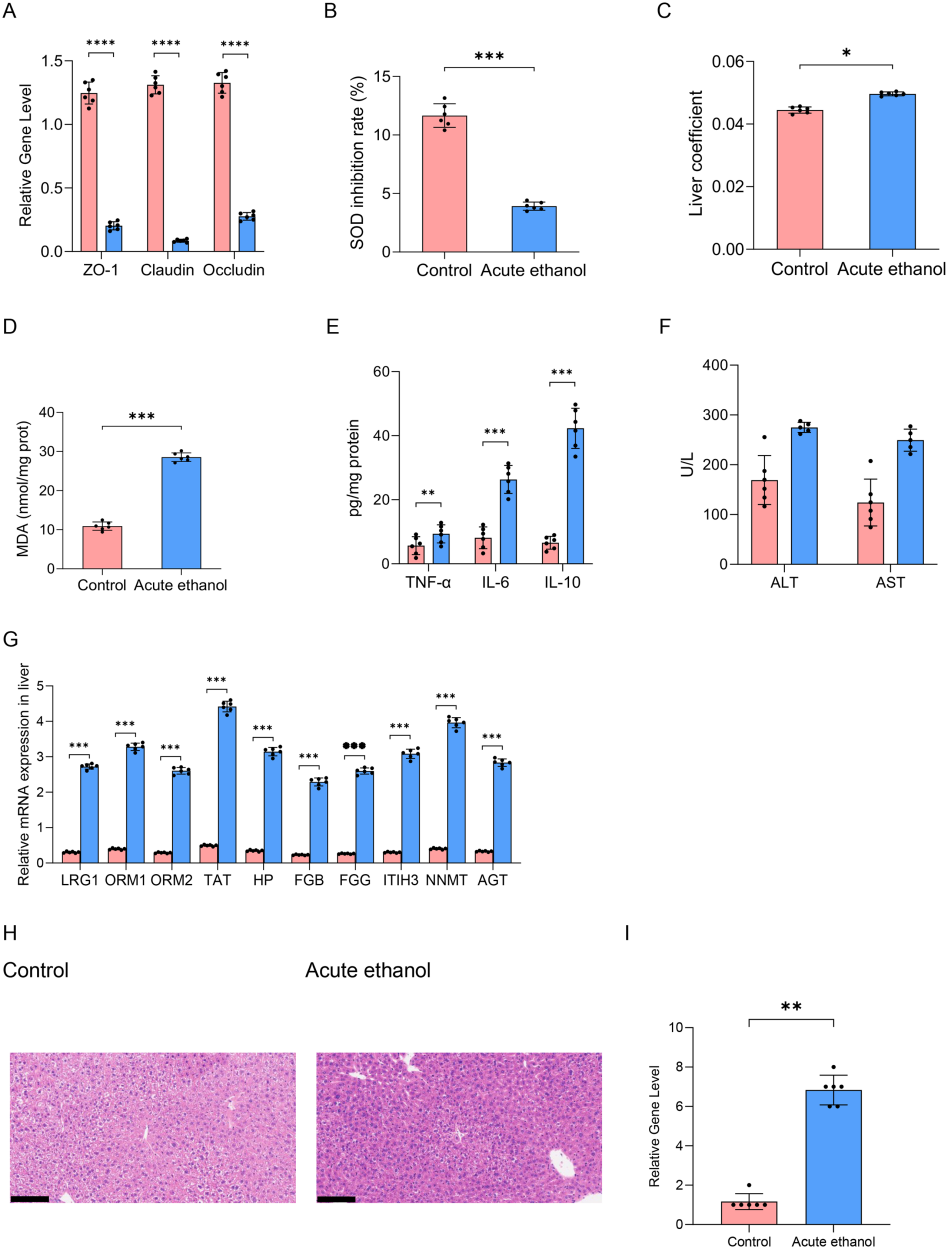
Acute ethanol exposure impairs intestinal barrier integrity and induces hepatic oxidative stress, inflammation, injury, and upregulation of hepatocyte-associated candidate genes in mice. Pink, Control; blue, acute ethanol. **(A)** Expression levels of intestinal tight junction markers ZO-1, Claudin-1, and Occludin in control and acute ethanol-exposed mice. **(B)** Hepatic superoxide dismutase (SOD) activity in control and acute ethanol-exposed mice. **(C)** Liver coefficient, calculated as the ratio of liver weight to body weight. **(D)** Hepatic malondialdehyde (MDA) levels as an indicator of lipid peroxidation. **(E)** ELISA measurement of IL-10 TNF-α, and IL-6 levels in liver tissues from control and acute ethanol-exposed mice. **(F)** Serum alanine aminotransferase (ALT) and aspartate aminotransferase (AST) levels in control and acute ethanol-exposed mice. **(G)** Quantitative real-time PCR analysis of Lrg1, Orm1, Orm2, Tat, Hp, Fgb, Fgg, Itih3, Nnmt, and Agt mRNA expression in liver tissues. **(H)** Representative hematoxylin and eosin **(H,E)** staining of liver sections from control and acute ethanol-exposed mice. **(I)** Semi-quantitative histological injury score based on H&E-stained liver sections. n = 6 per group for all mouse biochemical, ELISA, qPCR, and histological analyses unless otherwise indicated.” Scale bar = 100 μm. Statistical significance was defined as *P < 0.05, **P < 0.01, ***P < 0.001, and ****P < 0.0001.

To assess intestinal barrier alterations after acute ethanol exposure, we first examined the expression of tight junction markers. Compared with control mice, acute ethanol-exposed mice showed significantly reduced expression of ZO-1, Claudin-1, and Occludin, indicating impaired intestinal barrier integrity (**Figure 9A**). Consistently, macroscopic assessment of cecal contents showed dark yellow, viscous, semi-solid contents without an obvious alcoholic odor in control mice, whereas acute ethanol-exposed mice exhibited darkened, loose-to-watery contents with an alcohol fermentation-like odor. Semi-quantitative disturbance scores were also higher in the ethanol group (**Supplementary Table 9**), further suggesting acute ethanol-associated intestinal disturbance.

We next evaluated hepatic oxidative stress and injury-related phenotypes. Acute ethanol-exposed mice showed reduced hepatic superoxide dismutase (SOD) activity (**Figure 9B**), increased liver coefficient (**Figure 9C**), and elevated malondialdehyde (MDA) levels (**Figure 9D**), indicating impaired antioxidant defense, hepatomegaly, and enhanced lipid peroxidation. In parallel, hepatic concentrations of IL-10 TNF-α, and IL-6 were significantly increased in acute ethanol-exposed mice compared with controls (**Figure 9E**), supporting the presence of an *in vivo* inflammatory response. Serum ALT and AST levels were also elevated in acute ethanol-exposed mice (**Figure 9F**), indicating hepatocellular injury.

To determine whether the ten hepatocyte-associated candidate genes identified in our integrative analyses were transcriptionally upregulated under these pathological conditions, quantitative real-time PCR was performed using liver tissues from an independent cohort of alcohol-fed mice. All ten genes—Lrg1, Orm1, Orm2, Tat, Hp, Fgb, Fgg, Itih3, Nnmt, and Agt—were significantly upregulated in acute ethanol-exposed mice relative to controls (**Figure 9G**). Among them, TAT and NNMT exhibited the most pronounced increases, whereas FGB showed moderate upregulation, suggesting differential degrees of transcriptional responsiveness to alcohol-induced injury.

Histopathological examination by H&E staining revealed marked liver injury in acute ethanol-exposed mice, characterized by inflammatory cell infiltration, hepatocellular ballooning, and disruption of normal hepatic architecture, which was further confirmed by significantly increased semi-quantitative histological injury scores (p=0.0043, **Figures 9H, I**).

## Discussion

ALD is a multifactorial disorder involving metabolic dysregulation, inflammatory injury, and gut–liver axis perturbation (1, 2, 12–14). However, how alcohol-associated fecal microbial and metabolic alterations relate to hepatic transcriptional responses remains incompletely understood. In this study, we integrated fecal metagenomics, untargeted fecal metabolomics, mouse liver bulk RNA sequencing, and publicly available human hepatic transcriptomic datasets, identifying six convergent fecal metabolic pathways and a ten-gene hepatocyte-associated signature linked to alcohol-related liver injury.

The gut–liver axis has been widely implicated in ALD pathogenesis, particularly through microbial products, intestinal barrier dysfunction, and metabolite-associated inflammatory signaling (1, 2, 14). Our study adds to this field by showing that acute ethanol exposure was associated with coordinated changes in fecal microbial functional potential and fecal metabolite composition (15, 16). The six convergent pathways identified across metagenomic and metabolomic layers, including nucleotide metabolism, the PPP, HIS, GST, glycerophospholipid metabolism, and the PTS, are biologically related to redox balance, amino acid metabolism, one-carbon metabolism, membrane remodeling, and microbial substrate transport (17–21). These pathways may therefore represent candidate metabolic programs associated with alcohol-related liver injury. However, this interpretation should be cautious. Metagenomic profiling reflects inferred microbial functional potential rather than confirmed metabolic activity, and fecal metabolite profiles are not equivalent to portal vein metabolite composition. Because portal metabolites constitute the more direct biochemical exposure encountered by the liver, the present fecal multi-omics data cannot prove a gut-to-liver metabolite relay. Thus, our findings support pathway-level correspondence between fecal microbial and metabolic alterations, but do not establish that microbial metabolites causally drive hepatic transcriptional changes. Although Procrustes analysis showed a statistically significant global correspondence between metagenomic and metabolomic profiles (M2 = 0.1008, P < 0.001), this result should be interpreted as cross-omics co-variation rather than evidence of causal coupling between microbial function and fecal metabolite production (22, 23).

A second important finding is that several host-interpretable pathways among these convergent fecal multi-omics-associated pathways, including PPP, HIS, and GST, showed hepatocyte-enriched activity patterns in the analyzed human ALD scRNA-seq dataset. Importantly, PTS was not considered a host hepatocyte pathway and was therefore excluded from the human single-cell pathway activity analysis. This observation is consistent with the central metabolic role of hepatocytes and with previous evidence that oxidative stress, redox imbalance, and mitochondrial dysfunction contribute to alcohol-induced liver injury (24, 25). Nevertheless, hepatocytes are intrinsically the dominant metabolic cell type in the liver, and higher pathway scores in hepatocytes do not by themselves demonstrate ALD-specific activation. In addition, the GSE236382 dataset lacks healthy liver or non-ALD disease-control samples, and detailed clinical metadata, including fibrosis stage, inflammatory grade, MELD score, and other disease-severity variables, were not uniformly available for all patients. Therefore, we could not perform patient-level adjusted analyses to determine whether the observed hepatocyte-enriched pathway activities were independent of disease severity or inflammatory burden.

By integrating mouse liver transcriptomic changes with hepatocyte-associated features from human single-cell data, we identified a ten-gene signature consisting of LRG1, ORM1, ORM2, TAT, HP, FGB, FGG, ITIH3, NNMT, and AGT. These genes are involved in acute-phase responses, coagulation cascades, amino acid metabolism, secretory function, and systemic inflammatory regulation. Their consistent upregulation across acute ethanol-induced liver injury and human ALD/AH transcriptomic datasets suggests that they may reflect a conserved hepatocyte-associated injury response. At the same time, these genes should not be interpreted as specific drivers of a gut–liver mechanism. Many of them are also compatible with broader hepatic stress, acute-phase activation, or inflammatory injury. Thus, the ten-gene module is best described as a candidate hepatocyte-associated transcriptional signature related to alcohol-associated liver injury, rather than a validated disease-specific or causal gene program.

The exploratory molecular subtyping analysis further suggests that this ten-gene signature may capture transcriptional heterogeneity among patients with alcohol-associated liver injury. ALD spans a wide clinical spectrum, from steatosis and steatohepatitis to cirrhosis and severe alcoholic hepatitis (26). In this context, gene modules reflecting hepatocyte stress, metabolic adaptation, and inflammatory response may help describe biological variation across patient groups. In the external human cohort, consensus clustering based on the ten genes separated patients into two molecular subgroups with different metabolic pathway activities and clinical outcome distributions. However, this result should be regarded as exploratory. Because MELD score, fibrosis stage, inflammatory grade, and other clinical severity variables were not uniformly available for multivariable adjustment, we cannot exclude the possibility that the two molecular subgroups mainly reflect differences in disease severity rather than discrete biological subtypes. In addition, because the current analysis is based mainly on transcriptomic clustering and categorical outcome comparison, it does not establish prognostic utility or immediate clinical applicability. Larger cohorts with detailed clinical variables, disease severity indices, survival data, and multivariable validation will be required before this signature can be used for patient stratification (27, 28).

Several limitations should be emphasized. First, the newly generated mouse data were derived from a single-binge ethanol gavage model, which captures early ethanol-associated injury but does not fully reproduce chronic ALD or severe alcoholic hepatitis (15, 16). Second, fecal metabolomic and metagenomic profiles measured 24 h after high-dose ethanol gavage may partly reflect acute intestinal disturbance rather than stable microbial remodeling. Although reduced ZO-1, Claudin, and Occludin levels, macroscopic cecal content changes, and metabolomic QC information were added to improve interpretation, these data cannot fully exclude confounding from altered intestinal transit, stool consistency, food intake, hydration, or luminal dilution. Third, Procrustes analysis, pathway overlap, microbe–metabolite correlations, and associations between fecal metabolites and hepatocyte-associated genes indicate cross-omics co-variation but do not prove causality. Therefore, the identified pathways and ten-gene signature should not be interpreted as evidence of direct gut-to-liver mechanistic regulation. Fourth, the public GSE236382 scRNA-seq dataset lacks healthy liver or non-ALD disease-control samples, and detailed fibrosis stage, inflammatory grade, MELD score, and other disease-severity variables were not uniformly available, limiting disease-specific and patient-level adjusted interpretation of the hepatocyte-enriched pathway findings. Fifth, although the ROC analysis showed strong discriminatory performance, the present study did not include an entirely independent external validation cohort that was prospectively collected and analyzed separately from the discovery and public transcriptomic datasets. Thus, the diagnostic or stratification potential of the ten-gene signature should be considered preliminary and requires validation in independent cohorts with harmonized clinical annotation. Finally, the present study lacks direct hepatocyte-focused functional validation. Future studies using chronic ethanol models, portal metabolomics, microbiota manipulation, metabolite perturbation, primary hepatocytes, HepaRG cells, AML12 cells, and hepatocyte-specific *in vivo* perturbation systems will be required to determine whether candidate metabolites or the ten-gene module directly regulate hepatocyte metabolic and inflammatory programs.

In summary, this study provides a hypothesis-generating multi-omics framework linking fecal microbial functional potential, fecal metabolic alterations, and hepatocyte-associated transcriptional features in alcohol-related liver injury. The six convergent pathways and ten-gene signature identified here are best interpreted as candidate alcohol-responsive features detectable across acute ethanol-induced liver injury and human ALD/AH transcriptomic contexts. Further validation in chronic disease models, portal metabolomic profiling, hepatocyte-based functional systems, and independent disease-control cohorts will be necessary to clarify their causal relevance and potential clinical utility.

## Data Availability

The datasets presented in this study can be found in online repositories. The names of the repository/repositories and accession number(s) can be found in the article/**Supplementary Material**.
