# Supplementary material for: Multi-omics analysis identifies a hepatocyte-associated signature in alcohol-related liver injury
Source: Front Immunol. 2026 Jul 22;17:1844110. doi: 10.3389/fimmu.2026.1844110 (PMC13437270; doi:10.3389/fimmu.2026.1844110)
Supplement: Supplementary file 1 [file DataSheet1.pdf]

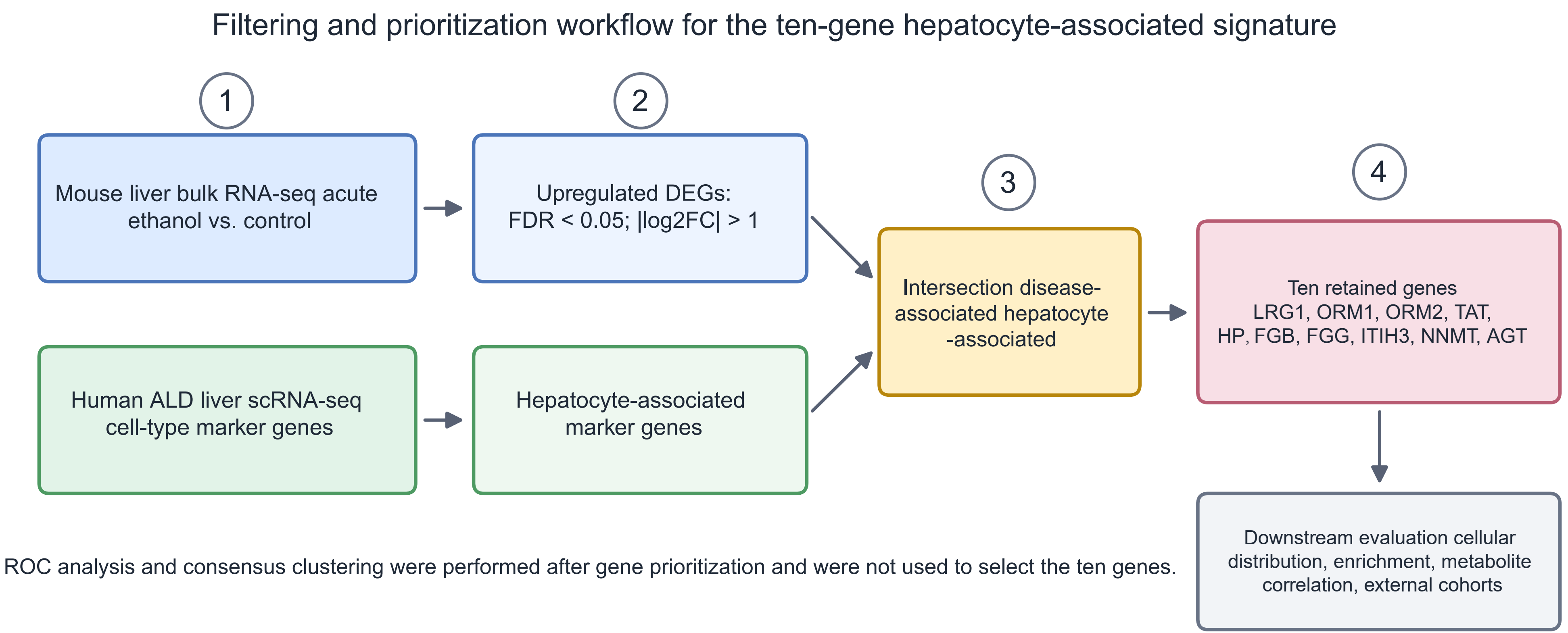
Filtering and prioritization workflow for the ten-gene hepatocyte-associated signature
1
2
3
4
Mouse liver bulk RNA-seq acute ethanol vs. control
Upregulated DEGs: FDR < 0.05; |log2FC| > 1
Human ALD liver scRNA-seq cell-type marker genes
Hepatocyte-associated marker genes
Intersection disease-associated hepatocyte -associated
Ten retained genes LRG1, ORM1, ORM2, TAT, HP, FGB, FGG, ITIH3, NNMT, AGT
Downstream evaluation cellular distribution, enrichment, metabolite correlation, external cohorts
ROC analysis and consensus clustering were performed after gene prioritization and were not used to select the ten genes.
